# Supplementary material for: Modeling chromatin state from sequence across angiosperms using recurrent convolutional neural networks
Source: Plant Genome. 2022 Aug 3;15(3):e20249. doi: 10.1002/tpg2.20249 (PMC12807023; doi:10.1002/tpg2.20249)
Supplement: Supplementary file 1 [file TPG2-15-e20249-s001.docx]

## Supplementary Information

Figure S1: Mean auPR of different across-species accessibility model configurations on the test set. From left to right: 1000 bp windows, 300 bp windows, 600 bp windows, training and testing within only grasses, using a training set balanced on both accessibility and distance class, exponential activation on the convolutional layer, and testing on Arabidopsis and Maize while training on the rest.

Figure S2: Receiver operating characteristic curves of the across-species models per hold-out species. The gray dashed line is the baseline expectation for a random classifier. The area under the curve is given inside the parentheses for each species in the figure legends.

Figure S3: Precision-recall curve comparison of the across-species a2z model with the bag-of-kmer model in *Z. mays*. The auPR is shown within parentheses in the figure legends.

Figure S4: auPR of both model configurations with respect to genome size. The dashed line is an exponential fit to the across-species model auPR values.

Figure S5: Performance comparison of different model architectures in the across-species accessibility model training configuration. Each colored line represents a different test species. In the left subfigure each point is the average performance of three models of a given architecture trained on all other species and tested within the unobserved species denoted by the point color. The right subfigure shows the difference in the average performance of each architecture from DanQ for each unobserved test species. The models are arranged on the x-axis by ascending average auPR across all test species.

Figure S6: Counts of accessible regions in the across-species test sets by distance class and species.

Figure S7: Comparison of false discovery rate (FDR) versus false omission rate (FOR) between models.

Figure S8: Precision-recall curve comparison between a2z model and the repeat-masked a2z model in *Z. mays*.

Figure S9: Precision-recall and receiver operating characteristic curves of an angiosperm-trained a2z model on yeast and a human cell line.

Figure S10: Cell type specificity of maize scATAC-Seq peaks.

Figure S11: Top 3 TF-MoDISco patterns for four a2z models. *A. thaliana* is the left column, *Z. mays* is the right column. Accessibility is the top row and methylation is the bottom row. Within each species and chromatin feature combination the patterns are ranked from top to bottom by the number of supporting seqlets for that pattern.

Figure S12: Proportion of high-effect kmers that significantly (q-value <= 0.05) matched JASPAR CORE *plantae* motifs with FIMO, grouped by species and chromatin feature.

Figure S13: Multidimensional scaling of the top 10 high-effect medoid kmers for each species and chromatin feature model combination, colored by species.

| **Accession** | **Species** | **Tissue** | **Library** | **Coverage (X)** |
| --- | --- | --- | --- | --- |
| SRX096372 | Arabidopsis thaliana | Leaf | 101bp SE | 81 |
| SRX1436235 | Eutrema salsugineum | Leaf | 101bp SE | 49 |
| SRX1656936 | Vitis vinifera | Leaf | 101bp SE | 28 |
| SRX1656930 | Populus trichocarpa | Leaf | 101bp SE | 31 |
| SRX200136 | Glycine max | Leaf | 101bp SE | 34 |
| SRX200137 | Glycine max | Leaf | 101bp SE | 27 |

Table S14: Data used to call dicot unmethylated regions.
